# Pathogenicity and Molecular Typing of Fowl Adenovirus-Associated With Hepatitis/Hydropericardium Syndrome in Central China (2015–2018)

**DOI:** 10.3389/fvets.2020.00190

**Published:** 2020-04-28

**Authors:** Jin Cui, Yingying Xu, Zutao Zhou, Qingrong Xu, Jiaxiang Wang, Yuncai Xiao, Zili Li, Dingren Bi

**Affiliations:** ^1^College of Veterinary Medicine, Huazhong Agricultural University, Wuhan, China; ^2^State Key Laboratory of Agricultural Microbiology, Huazhong Agricultural University, Wuhan, China; ^3^Key Laboratory of Preventive Veterinary Medicine in Hubei Province, Huazhong Agricultural University, Wuhan, China; ^4^College of Animal Science and Technology, Henan University of Animal Husbandry and Economy, Zhengzhou, China

**Keywords:** fowl adenovirus, fowl adenovirus (FAdV) serotype 4, molecular typing, pathogenicity analysis, China

## Abstract

In central China, a large number of broiler and layer flocks have suffered from outbreaks of severe hepatitis/hydropericardium syndrome (HHS). This resulted in huge economic losses to the poultry industry, from 2015 to 2018. To identify the specific pathogen and study its pathogenicity, 195 samples from Hubei, Jiangxi, Anhui, Hunan, and Henan provinces in central China were collected. The samples were screened for the adenovirus *hexon* gene, and neighbor joining was used for the phylogenetic reconstruction of the sequences. Among the collected samples, 122 were found to be positive for fowl adenovirus (FAdV) by PCR, and 73 isolates were obtained. The predominant viral serotype was serotype 4 (FAdV-4), which was found in 48 isolates, while 24 were serotype 10 (FAdV-10), and one was serotype 2 (FAdV-2). The CH/HBTF /1710 isolate was selected for further experiment and inoculated into 33-day-old specific pathogen-free chickens via intramuscular injection or oral administration to evaluate pathogenicity. It was found that the mortality for chickens infected by intramuscular injection or oral administration was 70 and 60%, respectively. Necropsy revealed mild to severe hepatitis and hydropericardium at 5 and 7 days after infection. Ancestor analyses indicated that all of the FAdV-4 strains obtained in this study shared a common Indian precursor and had a close genetic relationship with the JSJ13, SDSX, HN/151025, and SDDM-15 strains common in China.

## Introduction

Fowl adenoviruses (FAdVs) are non-enveloped viruses containing double-stranded linear DNA genomes and belonging to the genus *aviadenovirus*. FAdVs are grouped into five species (FAdV-A to FAdV-E) with 12 serotypes (FAdV-1 to −8a and −8b to −11) based on restriction enzyme digest patterns and serum cross-neutralization tests (1). The most notable diseases associated with FAdVs infection in chicken are the inclusion body hepatitis (IBH), hepatitis/hydropericardium syndrome (HHS), and gizzard erosions (GE) (2–4). All 12 serotypes of FAdVs have been associated with outbreaks of IBH, whereas HHS is primarily caused by FAdV serotype 4 (FAdV-4) strains, which has been classified as FAdV-C (5). IBH is mainly observed in 3- to 5-week-old broiler chickens, and the mortality approaches 10% (6). The main lesions of IBH are hepatic necrosis with microscopic eosinophilic or basophilic intranuclear inclusion bodies in hepatocytes (7, 8). HHS, also called hydropericardium syndrome (HPS), is an emergent, immunosuppressive disease of 3- to 6-week-old broilers, characterized by sudden onset and mortality from 30 to 70%, accumulation of clear or amber-colored liquid with aqueous or gelatinous consistency in the pericardial sac, enlarged, mottled, and friable liver with infiltration of mononuclear cells, and presence of basophilic intranuclear inclusions in hepatocytes (9–11).

HHS was first reported in Angara Goth, Pakistan, in 1987 and subsequent outbreaks have been recorded in many other countries, resulting in significant economic losses to poultry raisers in the United States, Canada, Germany, and Korea (5, 7, 10, 12). From 2015, severe HHS induced by FAdV-4 has emerged across several different areas in China, and clinical cases of HHS have been increasing since 2016 (11, 13). From 2015 to 2018, many cases of suspected FAdV infection occurred among broilers and layers in central China. Most of the cases showed severe HHS and caused high mortality resulting in tremendous economic losses to poultry farmers. The objective of the present study was to carry out the molecular typing of FAdV strains from HHS cases in Hunan, Hubei, Anhui, Jiangxi, and Henan provinces of central China and to study the pathogenicity of these strains in specific pathogen-free (SPF) chickens.

## Materials and Methods

### Eggs and Chickens

Specific pathogen-free (SPF) white nick chickens and embryonated eggs were obtained from Beijing Merial Vital Laboratory Animal Technology Co., Ltd (Beijing, China). The birds were maintained in isolators under negative pressure with *ad libitum* feed and water.

### Sample Collection and Treatment

From 2015 to 2018 in central China, liver samples were collected from broiler and layer chickens suspected of having HHS, characterized by pericardial effusion or hemorrhagic hepatitis. The tissue samples were homogenized in phosphate-buffered saline (PBS; 0.1 M, pH 7.2) at a ratio of 1:5. After three freeze–thaw cycles, the homogenates were centrifuged at 8,000 *g* for 20 min at 4°C. The supernatants were removed, passed through a 0.2-μm filter and immediately stored at −70°C until PCR analysis, and virus isolation was performed (11).

### FAdV Detection by PCR and Sequencing

Total DNA was extracted from supernatants using a DNA extraction kit (Invitrogen, Carlsbad, CA, USA) according to the manufacturer's instructions. The adenovirus *hexon* gene was used to study the taxonomy and antigenic properties of FAdVs (14). Based on the highly conserved region of the *hexon* gene, specific primers H1 (H1f, 5′-TGGACATGGGGGCGACCTA-3′, H1r, 5′-AAGGGATTGACGTTGTCCA-3′) and H2 (H2f, 5′-AACGTCAATC CCTTCAACCACC-3′, H2r, 5′-TTGCCTGTGGCGAAAGGCG-3′) were designed to amplify the complete coding sequence of the *hexon* gene of FAdV. PCRs were performed with the following parameters: 94°C for 5 min followed by 30 cycles of denaturation at 94°C for 1 min, annealing at 55°C for 1 min, and extension at 72°C for 1 min with a final extension at 72°C for 10 min. The PCR products were run on a 1.0% agarose gel. The reaction volume was 25 μl consisting of 1 μl (10 pmol) of each primer, 12.5 μl of Taq SuperMix (TaKaRa, Dalian, China), 2.5 μl of DNA, and 8 μl of nuclease-free water. The amplified products were cloned into the pMD18-T vector (TaKaRa, Dlian, China) for sequencing. After sequencing, the *hexon* genes were assembled using the Seqman program in the DNASTAR software package.

### Phylogenetic Analysis of the FAdV *hexon* Gene

The nucleotide sequences of the *hexon* genes were aligned with homologous sequences using the Lasergene sequence analysis software package (DNASTAR Inc., Madison, WI, USA) and were compared to reference sequences using MegAlign. The reference isolates included strains from the five species (FAdV A–E) with 12 serotypes (FAdV-1 through 7, 8a, 8b, and 9–11). A phylogenetic tree was constructed using the neighbor-joining method of MEGA version 5.2, and bootstrap *p*-values were determined from 1,000 replicates of the original data (15).

### Viruses Used for Experimental Infections

Samples that were positive by PCR were inoculated into monolayer cultures of 15-day-old SPF chicken embryo liver cells (CELC). Cultures of 5 × 10^5^ cells/dish (60-mm culture plates) were incubated at 37°C in 5% CO_2_. Aliquots of 0.2 ml of supernatant were pipetted onto CELC, and virus adsorption was allowed to take place at 37°C for 2 h. The culture supernatants were then discarded, and fresh Dulbecco's modified Eagle's medium supplemented with 2% fetal bovine serum was added to the CELC. Supernatants and cells were harvested after 48-h incubation, and three blind passages were performed. The determination of the median tissue culture infectious dose (TCID_50_) of the FAdV isolates in CELC was conducted according to a standard procedure, and TCID_50_ values ranged from 10^4.75^ to 10^7.5^. For infection, the CH/HBTF/1710 isolate was used at 10^7.5^/0.1 ml.

### Pathogenicity of FAdV Isolates in SPF Chickens

Seventy 33-day-old SPF white nick chickens were used in this study. The chickens were randomly divided into three groups with 20 birds per group. One group was infected with the CH/HBTF/1710 isolate by intramuscular injection, a second group was infected via the oral route, and the remaining 20 chickens were used as uninfected controls. The infectious dose was 10^7.5^ TCID_50_ per chicken in a volume of 0.2 ml, and the control chickens received the same volume of sterile PBS. In addition, 10 chickens were marked as contact sentinels and added to the oral route group given PBS only. The chickens were housed in isolators under negative pressure, observed twice a day and observed for clinical signs for 21 days. Clinical signs were scored as follows: 0 = normal, 1 = mild depression, 2 = severe depression, 3 = paralysis/prostration, and 4 = death (16). Dead chickens were necropsied to assess gross pathologic lesions, and samples of heart, liver, spleen, thymus, and kidney tissues were fixed in 10% neutral-buffered formalin for histology analysis.

### Histopathology and Immunohistochemistry

Samples of heart, liver, kidney, spleen, and thymus were fixed in 10% neutral formalin at room temperature for 24 h. After fixation, samples were processed, embedded in paraffin wax, and cut into 5-μm sections. The sections were dewaxed, stained with hematoxylin and eosin (HE), and observed by visible light microscopy. Paraffin block sections of heart, liver, kidney, spleen, and thymus were processed according to standard immunohistochemical (IHC) protocols with minor modifications. Briefly, sections were dewaxed in xylene and rehydrated with increasing concentrations of ethanol. Antigens were retrieved by immersing sections in 0.05% tween-20 and 0.01 mol/L pH 6.0 citric acid buffer for 20 min. Non-specific binding was blocked by incubation in 5% normal rabbit serum in PBS for 10 min. Slides were then incubated with rabbit anti-FAdV-4 hyperimmune serum (1:40 dilution in 0.01 M PBS, laboratory preservation) at 4°C overnight, followed by washing and incubation with goat anti-chicken IgG/Bio (ACROBiosystems, Beijing, China) for 30 min at 37°C and washed three times with phosphate buffer (0.05 M, pH 7.2). Immunocomplexes were detected using the 3,3-diaminobenzidine-enhanced liquid substrate system (TIANamp, Beijing, China), and the sections were counterstained with hematoxylin, air dried, and examined by visible light microscopy.

### Statistical Analysis

All analyses were performed using an independent-sample *t* test contained in the software SAS 9.3, and a value of *p* < 0.05 was considered statistically significant. Results are expressed as means ± standard deviation.

## Results

### Nucleotide Sequencing and Phylogenetic Analysis

**Figure 1:**
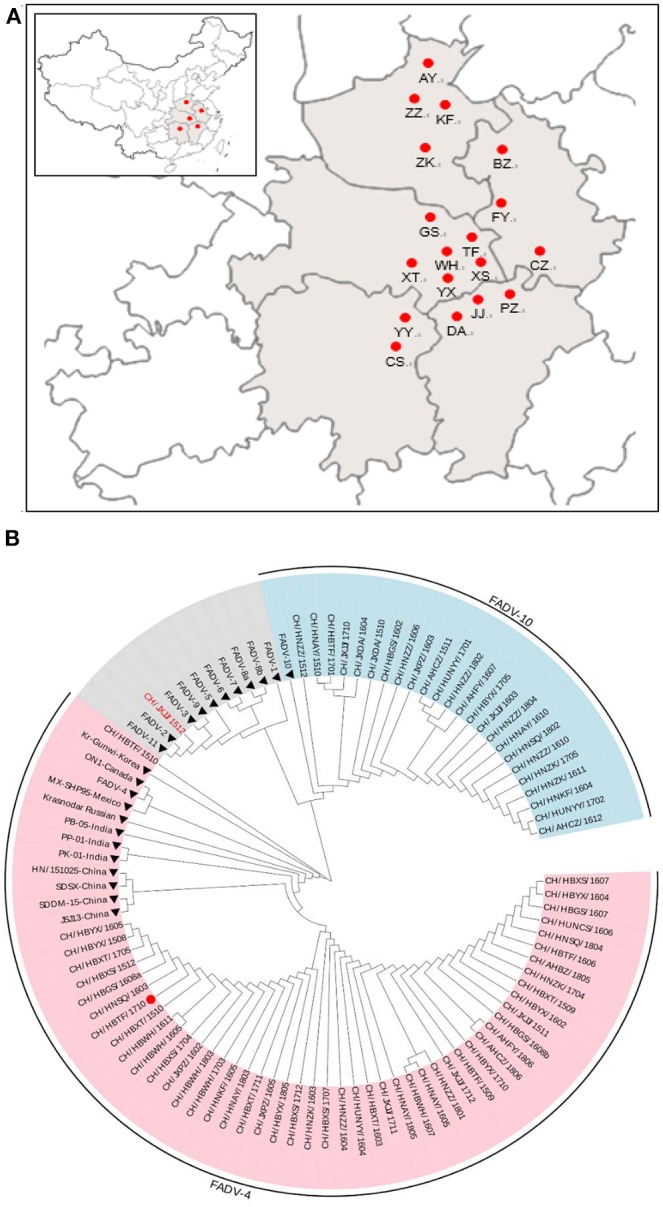
Geographical distribution of fowl adenovirus (FAdV) infection and phylogenetic analysis of isolates collected from different regions in central China from January 2015 to May 2018. **(A)** The Chinese provinces (in gray) and cities (red dots) where FAdV-infected chickens were collected in this study. **(B)** Phylogenetic analysis of the *hexon* gene from 73 isolates and 23 reference strains of FAdVs (filled diamonds) using MEGA version 5.2 with the neighbor-joining method and 1,000 bootstrap replicates. The red dot indicates the pathogenicity assessment of the CH/HBTF/ 1710 isolate.

A total of 195 clinical liver samples were collected from dead or diseased chickens (152 broilers, 43 layers) displaying HHS from broiler and layer flocks located in central China, in Hubei, Anhui, Jiangxi, Hunan, and Henan provinces (Figure 1A). A total of 122 samples (93 broilers, 29 layers) were PCR positive for FAdVs. Seventy-three unique FAdVs were isolated and sequenced including 57 from broilers and 16 from layers (Table 1). After assembly and verification, the sequences of the 73 FAdVs were submitted to GenBank (submission ID: 2262696). A phylogenetic analysis based on the obtained sequences was used to classify the 73 FAdVs into three serotypes. Forty-eight isolates of 89 positive samples were clustered in the FAdV-4 serotype sharing 68.07–97.18% nucleotide identity with *hexon* gene sequences of the FAdV-4 reference strain (GenBank accession No. HE608152). Twenty-four isolates from 32 positive samples were classified as FAdV-10, with 98.62–98.78% identity with the FAdV-10 reference strain (GenBank accession No. KT717889). The isolate, CH/JXJJ/1512, belonged to FAdV-2 and shared 99.84% nucleotide identity with the FAdV-2 reference strain (GenBank accession No. KT862805). The phylogenetic tree reconstruction is presented in Figure 1B.

**Table 1:** Information for the 73 FAdV isolates in this study.

**Province^a^**	**City^b^**	**FAdV isolates**	**Poultry type (age)^c^**	**Isolates date**	**Serotype**
Hunan (HUN)	Changsha (CS)	CH/HUNCS/1606	Broiler (32 days)	Jun 2016	FAdV-4
	Yueyang (YY)	CH/HUNYY/1604	Broiler (12 days)	Apr 2016	FAdV-4
		CH/HUNYY/1701	Layer (3 weeks)	Jan 2017	FAdV-10
		CH/HUNYY/1702	Broiler (24 days)	Feb 2017	FAdV-10
Anhui (AH)	Bozhou (BZ)	CH/AHBZ/1805	Layer (5 weeks)	May 2018	FAdV-4
	Fuyang (FY)	CH/AHFY/1607	Layer (19 weeks)	Jul 2016	FAdV-10
		CH/AHFY/1806	Broiler (50 days)	Jun 2018	FAdV-4
	Chizhou (CZ)	CH/AHCZ/1511	Broiler (32 days)	Nov 2015	FAdV-10
		CH/AHCZ/1612	Broiler (10 days)	Dec 2016	FAdV-10
		CH/AHCZ/1806	Broiler (15 days)	Jun 2018	FAdV-4
Jiangxi (JX)	Jiujiang (JJ)	CH/JXJJ/1511	Broiler (28 days)	Nov 2015	FAdV-4
		CH/JXJJ/1512	Broiler (22 days)	Dec 2015	FAdV-2
		CH/JXJJ/1603	Broiler (18 days)	Mar 2016	FAdV-10
		CH/JXJJ/1710	Broiler (12 days)	Oct 2017	FAdV-10
		CH/JXJJ/1711	Broiler (32 days)	Nov 2017	FAdV-4
		CH/JXJJ/1712	Broiler (35 days)	Dec 2017	FAdV-4
	Pengzhou (PZ)	CH/JXPZ/1602	Layer (15 weeks)	Feb 2016	FAdV-4
		CH/JXPZ/1603	Layer (20 weeks)	Mar 2016	FAdV-10
		CH/JXPZ/1605	Broiler (41 days)	May 2016	FAdV-4
	Dean (DA)	CH/JXDA/1510	Broiler (11 days)	Oct 2015	FAdV-10
		CH/JXDA/1604	Broiler (9 days)	Apr 2016	FAdV-10
Henan (HN)	Zhengzhou (ZZ)	CH/HNZZ/1512	Layer (38 weeks)	Dec 2015	FAdV-10
		CH/HNZZ/1604	Boiler (25 days)	Apr 2016	FAdV-4
		CH/HNZZ/1606	Broiler (23 days)	Jun 2016	FAdV-10
		CH/HNZZ/1610	Broiler (40 days)	Oct 2016	FAdV-10
		CH/HNZZ/1801	Broiler (32 days)	Jan 2018	FAdV-4
		CH/HNZZ/1802	Layer (30 weeks)	Feb 2018	FAdV-10
		CH/HNZZ/1804	Broiler (17 days)	Apr 2018	FAdV-10
	Anyang (AY)	CH/HNAY/1510	Layer (14 weeks)	Oct 2015	FAdV-10
		CH/HNAY/1605	Layer (12 weeks)	May 2016	FAdV-4
		CH/HNAY/1610	Layer (15 weeks)	Oct 2016	FAdV-10
		CH/HNAY/1803	Broiler (40 days)	Mar 2018	FAdV-4
		CH/HNAY/1805	Broiler (26 days)	May 2018	FAdV-4
	Zhoukou (ZK)	CH/HNZK/1603	Broiler (12 days)	Mar 2016	FAdV-4
		CH/HNZK/1611	Broiler (7 days)	Nov 2016	FAdV-10
		CH/HNZK/1704	Broiler (16 days)	Apr 2017	FAdV-4
		CH/HNZK/1705	Layer (25 weeks)	May 2017	FAdV-10
	Shangqiu (SQ)	CH/HNSQ/1603	Broiler (32 days)	Mar 2016	FAdV-4
		CH/HNSQ/1802	Broiler (25 days)	Feb 2018	FAdV-10
		CH/HNSQ/1804	Broiler (35 days)	Apr 2018	FAdV-4
	Kaifeng (KF)	CH/HNKF/1604	Broiler (19 days)	Apr 2016	FAdV-10
		CH/HNKF/1605	Broiler (12 days)	May 2016	FAdV-4
Hubei (HB)	Wuhan (WH)	CH/HBWH/1605	Broiler (10 days)	May 2016	FAdV-4
		CH/HBWH/1607	Broiler (18 days)	Jul 2016	FAdV-4
		CH/HBWH/1611	Broiler (22 days)	Nov 2016	FAdV-4
		CH/HBWH/1703	Broiler (14 days)	Mar 2017	FAdV-4
		CH/HBWH/1803	Broiler (9 days)	Mar 2018	FAdV-4
	Yangxin (YX)	CH/HBYX/1508	Broiler (10 days)	Aug 2015	FAdV-4
		CH/HBYX/1602	Broiler (15 days)	Feb 2016	FAdV-4
		CH/HBYX/1604	Broiler (30 days)	Apr 2016	FAdV-4
		CH/HBYX/1605	Broiler (32 days)	May 2016	FAdV-4
		CH/HBYX/1705	Broiler (14 days)	May 2017	FAdV-10
		CH/HBYX/1710	Broiler (18 days)	Oct 2017	FAdV-4
		CH/HBYX/1805	Broiler (38 days)	May 2018	FAdV-4
	Guangshui (GS)	CH/HBGS/1602	Broiler (22 days)	Feb 2016	FAdV-10
		CH/HBGS/1607	Layer (20 weeks)	Jul 2016	FAdV-4
		CH/HBGS/1608a	Layer (25 weeks)	Aug 2016	FAdV-4
		CH/HBGS/1608b	Layer (28 weeks)	Aug 2016	FAdV-4
	Xiaotao (XT)	CH/HBXT/1509	Broiler (15 days)	Sept 2015	FAdV-4
		CH/HBXT/1510	Broiler (38 days)	Oct 2015	FAdV-4
		CH/HBXT/1603	Broiler (23 days)	Mar 2016	FAdV-4
		CH/HBXT/1705	Broiler (12 days)	May 2017	FAdV-4
		CH/HBXT/1711	Broiler (40 days)	Nov 2017	FAdV-4
	Tuanfeng (TF)	CH/HBTF/1509	Broiler (29 days)	Sept 2015	FAdV-4
		CH/HBTF/1510	Broiler (17 days)	Oct 2015	FAdV-4
		CH/HBTF/1606	Broiler (10 days)	Jun 2016	FAdV-4
		CH/HBTF/1701	Layer (10 weeks)	Jan 2017	FAdV-10
		CH/HBTF/1710	Broiler (28 days)	Oct 2017	FAdV-4
	Xishui (XS)	CH/HBXS/1512	Layer (40 weeks)	Dec 2015	FAdV-4
		CH/HBXS/1607	Broiler (13 days)	Jul 2016	FAdV-4
		CH/HBXS/1704	Broiler (15 days)	Apr 2017	FAdV-4
		CH/HBXS/1707	Broiler (35 days)	Jul 2017	FAdV-4
		CH/HBXS/1712	Broiler (19 days)	Dec 2017	FAdV-4

a *Provinces in China where samples were collected*.

b *Cities in the provinces where samples were collected*.

c *Type and age (in days or weeks) of the chickens collected for FAdV detection from different chicken flocks in China*.

*CH, China; FAdV, fowl adenovirus*.

*CH/HBGS/1608a and CH/HBGS/1608b were isolated on the same day*.

### Clinical Signs and Gross Pathology

**Figure 2:**
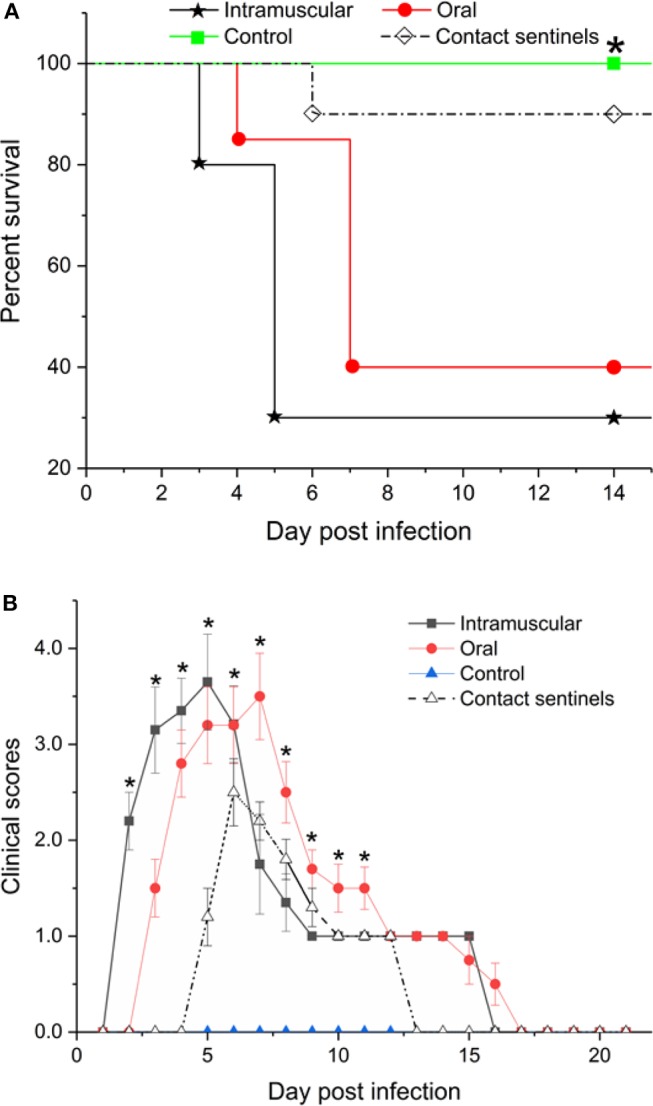
Survival rates **(A)** and clinical scores **(B)** of chickens inoculated with CH/HBTF/1710 by intramuscular injection and by the oral route. *(A) The percentages of birds surviving in the infected groups were significantly lower than in the control group (*p* < 0.05). **(B)** The clinical scores were significantly different between groups (*p* < 0.05).

Chickens inoculated with the CH/HBTF/1710 isolate by intramuscular injection showed obvious depression from 2 to 17 dpi. The first death occurred at 3 dpi, and at 5 dpi, mortality peaked. Four birds died at 3 dpi and 10 birds died at 5 dpi. In the oral infection group, chickens began to show clinical signs at 3 dpi, and 12 birds died from 4 to 7 dpi. All the contact sentinel birds began to show clinical signs at 5 dpi, and one chicken died at 6 dpi. All of the surviving chickens from the intramuscular and oral groups as well as the contact sentinels, gradually recovered from 7 dpi, and no further clinical signs were observed after 17 dpi. The survival rates of birds infected by intramuscular injection, oral infection, and contact were 30, 40, and 90%, respectively (Figure 2A). During the experimental period, different clinical scores were observed in the intramuscular injection, oral infection, and contact groups. The clinical scores for oral infection were less than those of intramuscular injection at 2–5 dpi (*p* < 0.05) but higher at 7–11 dpi (*p* < 0.05). The surviving chickens from the two infection groups gradually recovered from 7 to 9 dpi, chickens in the control group showed no clinical signs, and none died during the experiment (Figure 2B).

**Figure 3:**
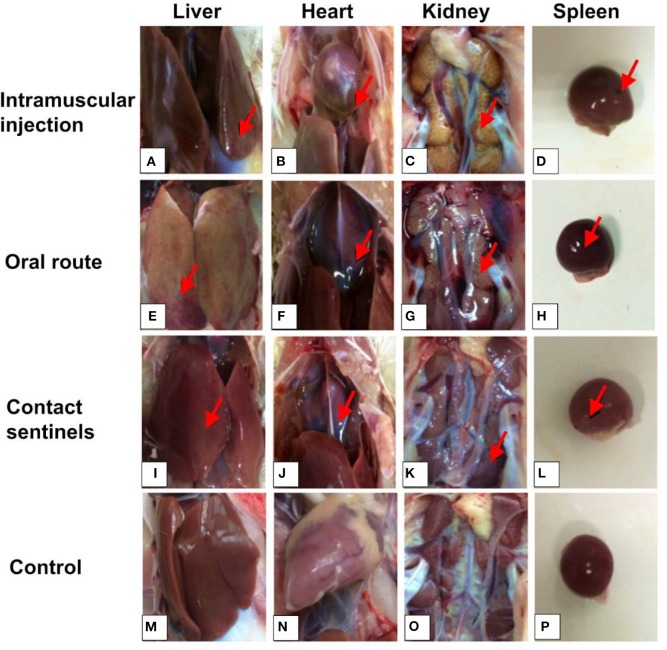
Gross examination of liver, heart, kidney, and spleen from dead chickens. **(A,E,I)** Swollen and friable liver with multifocal areas of necrosis. **(B,F,J)** Hemorrhage and hydropericardium in the heart. **(C,D,G,H,K,L)** Mild or severe enlargement in the kidney and spleen of the infected chickens. **(M–P)** Normal control. Arrow indicates the lesion area.

Necropsy examination of the birds at 5 and 7 dpi showed mild HHS, liver enlargement and friability, and pericardial effusion (Figures 3A,E,I). Hemorrhage and hydropericardium were detected in the heart (Figures 3B,F,J). Swelling of the kidneys and splenomegaly were also observed in all birds (Figures 3C,D,G,H,K,L). No significant gross lesions were present in the counterpart tissues of control chickens (Figures 3M–P).

### Histology and Immunohistochemistry

**Figure 4:**
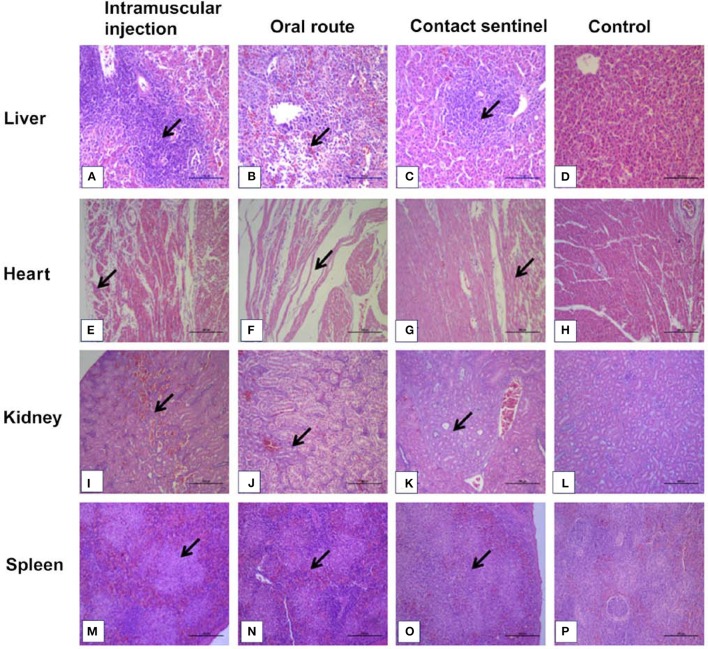
Histopathology in liver, heart, kidney, and spleen from dead chickens at 5 to 7 dpi (stained with HE) inoculated with CH/HBTF/1710. Massive pathological damage was observed in the virus-inoculated chickens. Degeneration, vacuolar necrosis, and basophilic inclusion bodies were present in hepatic cells **(A–C)**. Rupture and necrosis of myocardial fibers were observed in heart tissue **(E–G)**. Swelling and degeneration of renal tubular epithelial cells were present in the kidneys **(I–K)**. Severe reduction and necrosis of lymphocytes were seen in the spleen **(M–O)**. There was no significant histopathological damage present in the liver **(D)**, heart **(H)**, kidney **(L)**, and spleen **(P)** of chickens from the control group. Scale bar = 200 μm in **(A,B)**, and 100 μm in the other panels. Solid arrows point to lesion areas.

Tissue samples from the liver, heart, kidney, and spleen were collected from the different groups, fixed, paraffin-embedded, cut into sections, and stained with HE (Figure 4). No significant histologic lesions were noted in the non-infected control chickens (Figures 4D,H,L,P), while massive pathological damage was observed in tissues from the intramuscular injection group, the oral route group, and the contact sentinels.

**Figure 5:**
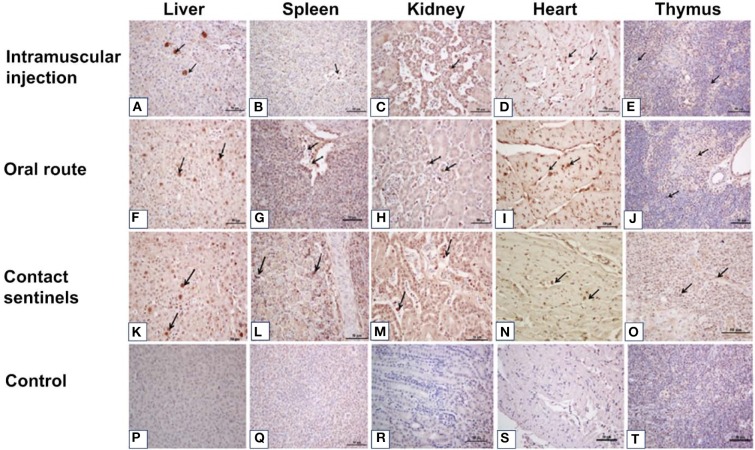
Immunohistochemical detection of FAdV antigens in the liver, spleen, kidney, heart, and thymus tissues after infection with FAdV CH/HBTF/1710 isolate at 5 to 7 dpi. Positive signals for FAdV antigen were extensively detected by immunohistochemical staining in the liver **(A,F,K)**, spleen **(B,G,L)**, kidney **(C,H,M)**, heart **(D,I,N)**, and thymus **(E,J,O)** in the two infection groups and the contact sentinels at 5-7 dpi. No FAdV antigen was detected in the liver **(P)**, spleen **(Q)**, kidney **(R)**, heart **(S)**, and thymus **(I)** in the control group. Solid arrows indicate positive signals for viral antigen. Scale bar = 50 μm.

The livers showed vacuolar degeneration and necrosis with basophilic inclusion in hepatic cells as indicated by solid arrows (Figures 4A–C). Disintegration and necrosis of myocardial fibers were observed in the heart tissue of infected chickens (Figures 4E–G). Extensive congestion in the renal interstitium and swelling and degeneration of renal tubular epithelial cells were observed in the kidneys (Figure 4K). Severe reduction and necrosis of lymphocytes was seen in the spleen (Figure 4O). Positive signals for FAdV antigen were extensively detected by immunohistochemical staining in the liver, spleen, heart, kidney, and thymus in the two infection groups and the contact sentinels at 5–7 dpi (Figures 5A–O). No FAdV antigen was detected in the control group (Figures 5P–T).

## Discussion

In recent years, an increasing number of IHB, HHS, and GE cases have been reported worldwide, and multiple FAdV strains have been isolated from avian species including ducks, chickens, and many wild as well as domestic birds (7, 8, 14, 17–19). IBH cases were first reported in 1986, in mainland China (20), but there were no reports of severe HHS prior to 2014 in China (16). However, since 2015, outbreaks of HHS have occurred with high mortality rates in chickens in most areas in China, resulting in enormous economic losses (11). In this study, 73 FAdV isolates in HHS-positive chickens from different regions in central China (Figure 1) were analyzed to determine phylogenetic relationships and virulence. Among the 73 FAdV isolates, 48 were identified and classified as serotype 4 by sequence alignment and phylogenetic analysis, 24 isolates were serotyped as FAdV-10, while CH/JXJJ/1512 was identified as FAdV-2. Interestingly, no sample in this study showed co-infection with FAdV-4, FAdV-10, and FAdV-2. Generally, FAdVs associated with IBH outbreaks were attributed to FAdV-8b, FAdV-7, and FAdV-11, while HHS cases were related to FAdV-4, based on *hexon* gene-sequence analysis. Among the FAdVs studied, 65.75% (48/73) were genetically related to serotype 4, further confirming that this serotype was the dominant agent of HHS in China from 2015 to 2018 (11, 13, 21–23). FAdV-10 strains were first detected in the Shandong province of China in 2017 (24). It is worth noting that 24 isolates detected in HHS chickens in our study were serotyped as FAdV-10, with 98.62–98.78% identity to strain C-2B (serotype FAdV-10). These results indicate that poultry farmers in China may be confronted with a risk of two serotypes epidemics in the future. Fortunately, FAdV-4 and FAdV-10 were classified as the same species of FAdV-C, and the fiber-1 proteins of the two serotypes, which directly bind to the viral receptor, are highly homologous. This makes it possible to develop a diagnostic tool for detection of FAdv- 4/10 (25).

**Figure 6:**
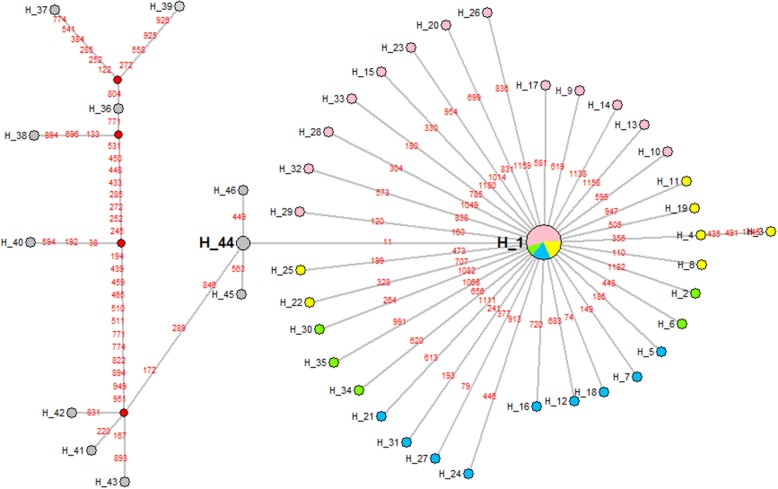
A maximum parsimony median-joining network of reference strains and the FAdV-4 isolates obtained in China in this study by Network version 5.2. Colors within the nodes refer to: reference strains (gray); the FAdV-4 strains isolated in China in 2015, 2016, 2017, and 2018, respectively (green, pink, blue, and yellow). Solid box: reference strains, the India strains PK-01, PP-01, and PB-05; dotted box: FAdV-4 isolates in China.

The phylogenetic tree of 48 *hexon* sequences from outbreak-associated FAdV-4 strains obtained in this study and 13 similar strains from GenBank was reconstructed using Network 5.2 software (Figure 6). The results showed that Chinese FAdV-4 strains including JSJ13 (H_44), SDSX (H_44), HN/151025 (H_45), and SDDM-15 (H_46) were clustered with the Indian strains, PB-05 India (H_41), PK-01 India (H_42), and PP-01 India (H_43). Interestingly, all Chinese sister clades shared a common Indian ancestor. The isolates of CH/HBES/171111, CH/HBXS/171202, CH/HNAY/180311, CH/AHBZ/180518, and CH/HBTF/1710 (H_1) formed a sister clade of JSJ13 and SDSX (H_44) strains. Therefore, this study indicates that the new strains in circulation in China after 2015 were derived from earlier Indian strains (21); however, the strains circulating in central China from 2015 to 2018 exhibited closed relations with JSJ13 and SDSX. To our knowledge, this is the first complete report about the origin of FAdV-4 ancestors in central China in recent years.

The pathogenicity of the CH/HBTF/1710 isolate was determined by observing the clinical signs, and the gross and histological lesions. The results demonstrated that the strain causing high mortality among 33-day-old SPF layer chickens and all birds that succumbed to infection showed serious HHS. Chickens infected by the intramuscular route showed high clinical scores and 70% mortality, which was consistent with the findings of previous studies (11, 13, 16). With respect to oral inoculation, the mortality approached 60%, which was higher than that obtained in previous studies (11, 26). The results indicated that the CH/HBTF/1710 isolate was possibly more virulent than other strains of serotype 4, even though the outcome of experimentally induced HHS may be related to genetic differences among strains within the same serotype, the route of infection, or differential susceptibility of the chickens (6). The contact chickens presented typical HHS symptoms, and death occurred among those housed with the orally infected group suggesting that HHS is contagious and can be transmitted horizontally among chickens (5). Histopathological changes were observed in the heart, kidney, and spleen, but particularly in the liver, which revealed the presence of intranuclear inclusion bodies and multifocal necrosis. Viral antigens were detected in almost all tissues sampled from necropsied animals.

## Conclusions

The results of this study showed that the FAdV-4 and FAdV-10 serotypes were involved in the outbreaks of HHS in central China from 2015 to 2018, and that the FAdV-4 serotype was the predominant one. The FAdV-4 isolates were clustered with JSJ13 and SDSX and shared an immediate common ancestor of an Indian strain. Infection of SPF chickens with the isolate CH/HBTF/1710 (FAdV-C, FAdV- 4) caused HHS and resulted in a high mortality of 70%. Collectively, this study clarified the origin of FAdV-4 strains and demonstrated the genetic epidemiology of FAdV circulating in China from 2015 to 2018. These data should help researchers to develop vaccines against multiple adenovirus serotypes to control and prevent the HHS disease among domestic chickens.

## Data Availability Statement

The datasets generated for this study can be found in NCBI GenBank, NCBI Accession No. MT121992-MT122064.

## Ethics Statement

Animal procedures and usage in this study were evaluated and approved by the Institutional Animal Experiment Committee from the Veterinary Faculty of Huazhong Agricultural University, Wuhan, China.

## Author Contributions

ZZ and DB designed research. JC and YX performed research. QX, JW, YX, and ZL contributed analytic tools. JC and ZZ analyzed data. JC wrote the paper.

## Conflict of Interest

The authors declare that the research was conducted in the absence of any commercial or financial relationships that could be construed as a potential conflict of interest.
